# Conditional robustness analysis for fragility discovery and target identification in biochemical networks and in cancer systems biology

**DOI:** 10.1186/s12918-015-0216-5

**Published:** 2015-10-19

**Authors:** Fortunato Bianconi, Elisa Baldelli, Vienna Luovini, Emanuel F. Petricoin, Lucio Crinò, Paolo Valigi

**Affiliations:** Dept of Experimental Medicine, University of Perugia, Polo Unico Sant’Andrea delle Fratte, Via Gambuli, 1, Perugia, 06156 IT; Center for Applied Proteomics and Molecular Medicine George Mason University, 10900 University Blvd, Manassas, 20110 USA; Dept of Medical Oncology, Santa Maria della Misericordia Hospital, Azienda Ospedaliera di Perugia, Piazzale Menghini, 1, Loc. Sant’Andrea delle Fratte, Perugia, 06156 IT; Dept of Engineering, University of Perugia, G. Duranti, 93, Perugia, 06125 IT

**Keywords:** Robustness analysis, Cancer robustness, Target therapies, Lung cancer, Drug discovery, Cancer systems biology, EGFR-IGF1R networks

## Abstract

**Background:**

The study of cancer therapy is a key issue in the field of oncology research and the development of target therapies is one of the main problems currently under investigation. This is particularly relevant in different types of tumor where traditional chemotherapy approaches often fail, such as lung cancer.

**Results:**

We started from the general definition of robustness introduced by Kitano and applied it to the analysis of dynamical biochemical networks, proposing a new algorithm based on moment independent analysis of input/output uncertainty. The framework utilizes novel computational methods which enable evaluating the model fragility with respect to quantitative performance measures and parameters such as reaction rate constants and initial conditions. The algorithm generates a small subset of parameters that can be used to act on complex networks and to obtain the desired behaviors. We have applied the proposed framework to the EGFR-IGF1R signal transduction network, a crucial pathway in lung cancer, as an example of Cancer Systems Biology application in drug discovery. Furthermore, we have tested our framework on a pulse generator network as an example of Synthetic Biology application, thus proving the suitability of our methodology to the characterization of the input/output synthetic circuits.

**Conclusions:**

The achieved results are of immediate practical application in computational biology, and while we demonstrate their use in two specific examples, they can in fact be used to study a wider class of biological systems.

**Electronic supplementary material:**

The online version of this article (doi:10.1186/s12918-015-0216-5) contains supplementary material, which is available to authorized users.

## Background

Most diseases, including cancer, involve a large number and variety of elements that interact via complex networks and, consequently, display highly nonlinear behavior. Traditional approaches to the study of biological phenomena consider single events, such as single mutations, single gene or protein alterations, and their corresponding effects on the biological phenotype. These reductionist approaches are intrinsically unable to fully capture the overall complexity of molecular interaction networks. The key main of systems biology is to take into account all the interactions within a given network, as emphasized by the *system* notion [1, 2].

The identification of specific molecular targets that play a central role in cancer cell proliferation and survival has led to the development of a targeted therapy approach for the treatment of cancer patients in the clinical setting. Nevertheless, knocking out one target molecule in a biochemical pathway may not be enough to treat a disease such as cancer, because tumor cells often find alternative molecular routes to escape the drug-induced blockage. This is one reason why current drug design strategies fail in some cases [3].

In the last few years, Systems Biology has received increasing attention as a promising approach toward personalized medicine and to assist the oncologist community [4]. Through the Systems Biology approach, for example, it could be possible to improve our understanding of the complex signalling networks involved in cancer. This methodology would allow for the development of smarter therapeutic strategies; e.g., disrupting simultaneously two or three key intersections crucial for tumor growth and progression in a biochemical network. This approach could lead to significant advances in the treatment of cancer and help in transforming traditional reductionism-based methods into systems-level ones for drug discovery [5–8].

The analysis of complete (or, at least, a large portion of) networks could be a way to understand the robustness property of cancer [9, 10]. In this study, we focus on cancer robustness as a quantitative measurement indicating the cells ability to maintain their functions against internal and external perturbations. In the framework of cancer research, it is relevant to discover how to reduce robustness of cell proliferation attitude; i.e., it is important to study the *fragility* of cancer cells and to discover ways to increase this fragility.

**Fig. 1:**
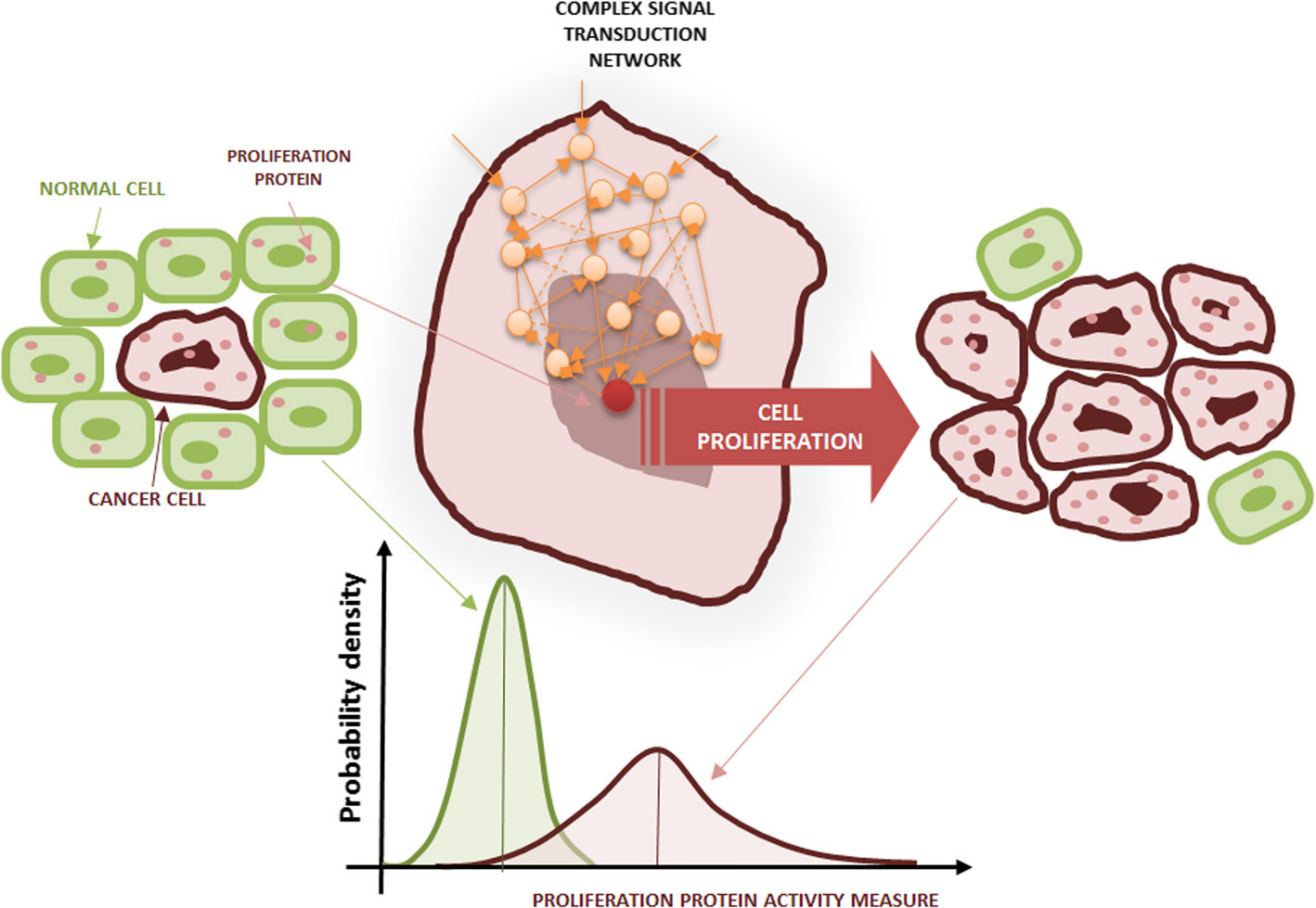
Cancer cell proliferation. Green cells are normal cells and red cells are tumor cells. The proliferation activity of normal and tumor cells can be measured looking at the activation of a proliferation protein, which is driven by a complex network based on protein interactions. In a population of cells the proliferation activity can be described by means of probability density for the proliferation protein; e.g., phosphorylated form of the extracellular signal-regulated kinase (ERK). The plots at the bottom show an example of probability density of a proliferation indicator in tumor (red line) and normal cells (green line), respectively

Figure 1 shows the context of interest of this paper. Signal transduction pathways are complex networks based on protein interactions that determine the propagation of extracellular inputs through the cytoplasm driving the timing of cellular survival, apoptosis and proliferation.

The proliferation activity of normal and tumor cells can be evaluated by looking at the activation of downstream proteins; e.g., phosphorylated form of extracellular signal-regulated kinase (ERK) [11]. To achieve quantitative measure of the proliferation activity, a suitable *proliferation indicator* can be used. For example, the intensity of phosphorylated ERK is one such indicator. If we describe the proliferation activity of a cell population by means of the probability density for the chosen proliferation indicator, the expected mean value and variance for cancer cells will be higher than normal cells. The plots at the bottom of Fig. 1 show an example of probability density of a proliferation indicator in tumor (red line) and normal cells (green line), respectively. Additional variability in the signaling networks, such as different topologies and settings, arises depending on cell type (e.g., lung, breast, colon,..) and their characteristics (normal vs cancer cell). The variability on drugs response can be seen as example [12].

To prove the suitability of our approach in a more general context, we investigated both an oncological network and a synthetic biology example. Synthetic biology aims to provide a way to synthesize living systems according to certain design specifications by means of strategies similar to the ones electrical engineers employ to construct electronic circuits[13]. Among the proposed applications of synthetic biology, there are medical applications, such as synthesis of biofuels, biosensing, selective destruction of cancerous cellular tissue and low-cost production of drugs [14]. At the heart of this new field are synthetic gene networks. Such circuits are frequently constructed in *Escherichia coli* by cutting and combining natural or engineered coding DNA regions and promoters. Widely studied examples of such circuits include a toggle switch [14], an auto-repressed circuit [15], an oscillating “repressilator” [16], as well as other electronics-inspired genetic devices, including pulse generators [17], digital logic gates, filters and communication modules [14].

Biochemical signal transduction networks, both natural and synthetic, can be modeled with a large spectrum of mathematical tools; here we focus on Ordinary Differential Equations (ODE) that allow us to describe the time evolution of a set of proteins of interest. Once the pathway structure is drawn, the corresponding equations are relatively easy to write down using widely accepted kinetic laws, such as the law of mass action, the Michaelis-Menten law or the Hill functions. The generated model will depend on several parameters, and the corresponding identification and model selection problems are relevant issues (see [18, 19] and the references therein).

The alterations giving rise to tumor development can be described by perturbations on the parameters characterizing the biochemical reactions; i.e., the parameters characterizing the whole set of ODE [20]. The lack of parameter identifiability in large-scale network models hamper translation of the results of modelling studies into the process of anti-cancer drug development. Hence, assuming large perturbations on those parameters and studying the corresponding Global Sensitivity Analysis (GSA) is a way to analyze the uncertainty of the model parameters and to generate valid predictions on parametric sensitivities [21, 22]. In [23] the authors applied GSA to an ErbB signalling network model with the goal of exploring how multi-parametric network perturbations affect signal propagation through cancer-related networks.

Other approaches to robustness analysis are those searching for the shape and volume of the region in the parameter space in which the system is functioning properly [24], and for the topology and geometry of such a region, which turns out to have important consequences for the robustness [25–27]. The topological properties of the networks and their relationship with robustness and fragility in large-scale bio-molecular networks have been studied [28], showing that networks with a larger number of positive feedback loops and a smaller number of negative feedback cycles are likely to be more robust against disturbances.

A related problem is model validation. In this case, a robustness index can be seen as a measure of plausibility in models of biochemical networks [29]. Along this line, [30] introduces the concept of “glocal” robustness analysis as a combination of global and local tools used for model validation and for identifying key causes of high or low robustness. A possibly related problem, studied in [31–33], is the core prediction.

The purpose of the present contribution arises from personalized therapy and is focused on computational analysis. We use the *in silico* model to generate samples of the proliferation indicator and some computational elaborations to select a small number of nodes in the cancer cells signaling network that reduces their proliferation robustness; i.e., that improves the *fragility* of cancer cell. It follows that these nodes are candidate drug targets and our algorithm permits us to discover how these nodes can be *conditioned* to obtain the desired behavior.

**Fig. 2:**
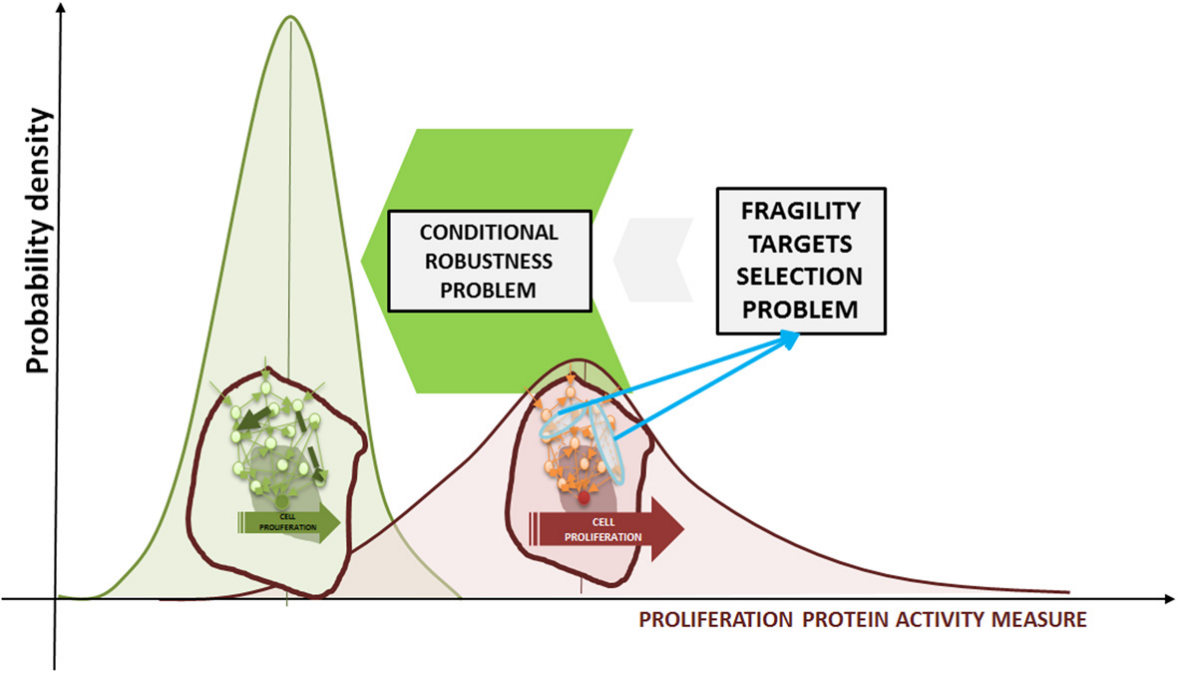
Problem formulation. The *fragility problem* in the oncology context is related to the cancer cells signaling network, and the goal is to reduce the cell proliferation attitude acting in few target molecules. The problem is related to the *conditional robustness problem*, namely the problem of shifting the probability density function of a proliferation signal in cancer cells toward the density describing the normal cells

Figure 2 illustrates the fragility problem: we propose a methodology to shift the probability density function of a proliferation signal in cancer cells toward the density describing the normal cells. The solution proposed here stems from robustness analysis tools, extending our previous results in [34]. Several authors have introduced the visionary idea of applying robustness in drug development [9, 10, 23, 35], although the application of the general definition is still a key problem in Systems Biology.

The rest of the paper is organized as follows. In the “Methods” section we introduce the complete theory associated with our procedure, namely the robustness problem, the conditional robustness concept, its use in the solution of the fragility problem, and the proposed computational algorithm. In the “Results” we illustrate the procedure with two examples drawn from molecular biology. The first example is a circuit from synthetic biology, the pulse generator circuit and the second one is a signal transduction network from Cancer Systems Biology. Finally, in the “Discussion and Conclusions” we summarize the new procedure, we give some additional remarks, and we point out how these findings should be of immediate interest to researchers in computational biology applied to cancer drug development.

## Methods

### Problem formulation

In this paper, we start from the general definition of *robustness* proposed by Kitano [36]: *robustness* is a property that allows a system $\mathcal {S}$ to maintain its property/capability *τ* against internal and external perturbations *p*. Kitano [36] proposes the following measure:

(1) $$ R^{\mathcal{S}}_{\tau,\mathbb{P}} := \int_{\mathbb{P}} \psi(p) \zeta^{\mathcal{S}}_{\tau}(p) dp,   $$

where *ψ*(*p*) is the probability of parameter vector *p*, $\mathbb {P}$ is the parameter space and $\zeta ^{\mathcal {S}}_{\tau }(p)$ is the evaluation function of capability *τ* for the system $\mathcal {S}$. Kitano’s definition is very general and can be used in a wide number of applications and problems.

The evaluation function $\zeta ^{\mathcal {S}}_{\tau }(\cdot)$ for the systems $\mathcal {S}$ can be considered as an input-output map relating the parameters *p* to the function $z, z \in \mathbb {R}$, measuring the capability of interest *τ*:

(2) $$ \zeta:\mathbb{P} \longrightarrow \mathbb{R}, \quad z=\zeta^{\mathcal{S}}_{\tau}{(}{p}{)}.  $$

The function $\zeta ^{\mathcal {S}}_{\tau }(p)$ is not necessarily known analytically, and in Systems Biology applications it is often computed *in silico* through a simulation of the mathematical model of system $\mathcal {S}$ for a given value *p* of the parameter vector. In this paper we will assume that the biological system $\mathcal {S}$ can be modeled by a system of ordinary differential equations of the form:

(3) $$ \mathcal{S} : \left\{\begin{array}{rcl} \dot{x} & = & f(x,u,p), \quad x(0)=x_{0}, x \in \mathbb{R}^{n}, \; p \in \mathbb{P} \\ z & = & h(x,p), \quad y \in \mathbb{R}^{m} \end{array}\right.   $$

where the state space vector *x* denotes the concentration of some substances relevant to the biological phenomena under consideration; *p* denotes the vector of system parameters taking values in the *parameter space*$\mathbb {P}$, a subset of the positive orthant $\mathbb {R}_{>0}^{q}$ of $\mathbb {R}^{q}$; *u* denotes the input vector; i.e., the external stimuli acting on the system; and *z* denotes the output response of the system; e.g., the evaluation function of interest.

### Conditional robustness

#### Conditional robustness: short motivation

In several applications, and in Systems Biology as a special case, it is of interest to search for a (possibly small) subset of the parameter vector having strong influence on the evaluation function $\zeta ^{\mathcal {S}}_{\tau }(\cdot)$, and allowing the system to exhibit extreme values for it.

For example, in the case of translational oncology and targeted therapy [6–8], the idea is that one has to carefully choose a *few nodes* along the pathway where to act pharmacologically, with the aim of achieving the *best possible benefit* [9, 10, 37]. Also in Synthetic Biology, the fine-tuning through a few parameters of a synthetic network is searched for to produce the input/output behaviors characteristic that can be used as the data sheet of biological circuits [14].

To this purpose, we introduce the notion of *conditional robustness* as follows.

#### Density probabilities on *P* and *Z*

We can consider a given vector *p* in the parameter space as a realization of a vector random variable *P* on a measurable space $(\mathbb {P},\mathcal {A})$. We denote by *F*_*P*_(*p*) the cumulative distribution function of *P*, which is a model of the “a priori” knowledge on *P*. Let *f*_*P*_(*p*) denote the corresponding probability density function.

Similarly, the evaluation function $\zeta ^{\mathcal {S}}_{\tau }(\cdot)$ can be interpreted as a transformation from the random variable *P* to the random variable *Z*, whose realizations are given by $z=\zeta ^{\mathcal {S}}_{\tau }(p)$. Hence, let *F*_*Z*_(*z*) and *f*_*Z*_(*z*) denote the cumulative distribution function and the probability density function of the model output *Z*, respectively, that is, the transformation of the corresponding measures *F*_*P*_(*p*) and *f*_*P*_(*p*) through the mapping *ζ*.

#### Partitioning definition domain *Z*

**Fig. 3:**
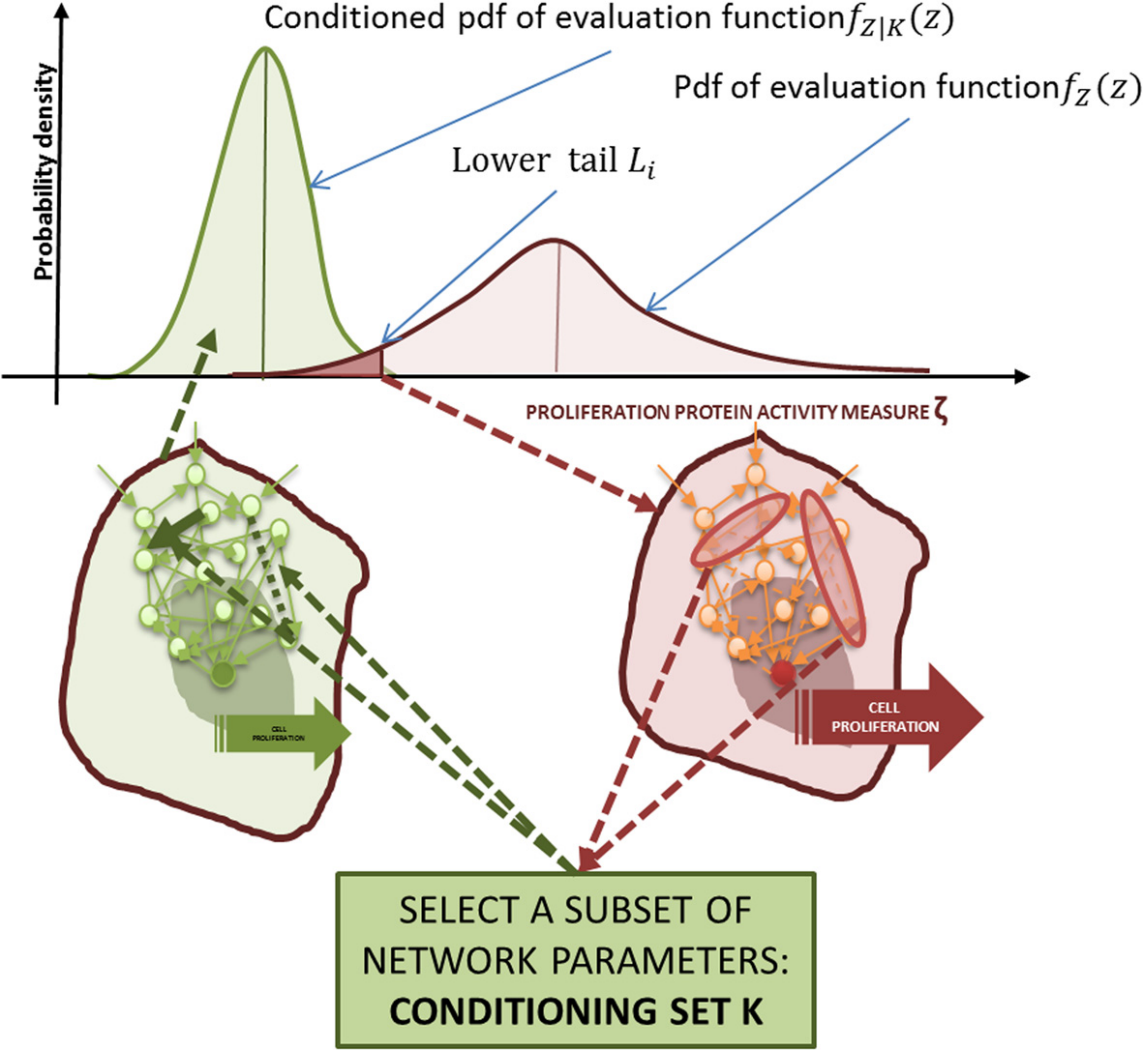
General problem formulation of conditional robustness. The problem addressed in this paper is that of selecting a suitable *conditioning set* *K* (a proper subset of parameter vector and the corresponding values), achieving a conditional probability density function *f*_*Z*|*K*_(*z*) having values of the output function in a given lower set *L*_*i*_

The system behavior, in addition to the output *Z*, can also be characterized by means of the above mentioned density function *f*_*Z*_(*z*) and by means of the associated probability of having values of the measure *Z* within a given interval of its definition domain. The red curve in Fig. 3 is an example of probability density function of the model output; i.e., the proliferation activity in a population of cancer cells.

To further characterize system behavior, we consider some subset *T*(*α*) of the definition domain $\mathcal {D}_{Z}$ of the output *Z*. For example, in the case of the normal distributions, examples of subsets *T*(*α*) are the *lower quartile* and the *upper quartile*. Here, we will use a subset *T*(*α*) defined according to one of the following conditions:

(4a) $$\begin{array}{@{}rcl@{}} T =T(\alpha)&:=& \left\{ z \le a : {\int_{0}^{a}}f_{Z}(z)dz=\alpha\right\},  \end{array} $$

(4b) $$\begin{array}{@{}rcl@{}} T =T(\alpha)&:=& \left\{ z \ge a : \int_{a}^{\infty}f_{Z}(z)dz=\alpha\right\}. \end{array} $$

Notice that the above definitions assume that the output function spans the non negative real axes. We will call the class of subsets of type (4a) the “lower set”, and the ones given by (4b) the “upper set”, and we will also use the notation *L*=*L*(*α*) and *U*=*U*(*α*), respectively, to make it clearer. In Fig. 3 the lower set for the proliferation indicator is marked with dark red color under the *f*_*Z*_(*z*) curve.

More generally, we can define a set *T*(*α*,*a*,*b*) such that:

(5) $$\begin{array}{*{20}l} T(\alpha, a, b) :&=\left\{{\vphantom{{\int_{a}^{b}}}} z\in \mathcal{D}_{Z} : Pr\{a<Z<b\}\right.\\ &=\left.{\int_{a}^{b}}f_{Z}(z)dz<\alpha\right\}.  \end{array} $$

Of course, the three values *a*, *b* and *α* in (5) are not independent.

A different approach to the partitioning of the output space $\mathcal {D}_{Z}$ is based on population quartile; i.e., by collecting a fixed number of samples, say the 10 *%* of samples, with the lower values of the evaluation function. Numerical experiments with such an approach, not reported here for brevity, yield results pretty much equal to the ones achieved with the probability approach proposed in (4).

#### Conditioning on *P*_*i*_

Suppose now that we choose to fix the *i*-th component of the parameter vector at a given value $\hat {p_{i}}$. We introduce the notion of *conditional robustness* of system $\mathcal {S}$ given that the scalar variable *P*_*i*_ is fixed at $\hat {p_{i}}$. It will be studied by means of the conditional probability on the output *Z*; i.e., the probability density function $f_{Z|P_{i}}(z)$ of *Z* upon selection of *P*_*i*_ to a given value $\hat {p_{i}}$. An example of conditional density distribution is shown in Fig. 3 with a green line.

#### The problem: qualitative formulation

With the above notation, the **problem studied in this paper** is that of selecting a suitable *conditioning setK* (a proper subset of parameter vector and the corresponding values), achieving a conditional probability density function *f*_*Z*|*K*_(*z*) properly placed on the support interval shared with the density *f*_*Z*_(*z*) (Fig. 3). With “properly placed” we mean, as an example, that the conditional probability of having values of the output function in a given (upper or lower) set *T* has to be much larger than the probability of the same set in the unconditioned case. This allows for a moment independent formulation of our problem (again, see Fig. 3).

#### The problem name: fragility problem

Taking into account that our main interest originates in the oncological context, and more precisely in reducing the cell proliferation attitude, we will call such a problem the *fragility problem*.

### Solution to the fragility problem

#### The proposed solution: conditional density $f_{P_{i}|T}(p_{i})$

The proposed approach to the solution of the fragility problem relies on the availability of the probabilities $f_{P_{i}|T}(p_{i})$ of each parameter *P*_*i*_, interpreted as a random variable, conditioned by the values of the output function *Z* belonging to a given subset *T* of the output space. Assuming the definitions (4) for such a subset, we use the density $f_{P_{i}|T}(p_{i})$ to characterize the portion of the parameter space along the direction *P*_*i*_ giving rise to the values of the output function belonging to such set *T*.

### The proposed solution: selection of set *K*

In order to select a conditioning set *K*, the problem is now to search for the directions in the parameter space giving a sufficiently sharp separation of the conditional probabilities $f_{P_{i}|L}(p_{i})$ and $f_{P_{i}|U}(p_{i})$; i.e., the set *K* which has a stronger influence on the system output *Z*; i.e., on the evaluation function of interest. The basic idea is to compute for each of the components of the parameter vector the two conditional densities, $f_{P_{i}|U}(p_{i})$ and $f_{P_{i}|L}(p_{i})$. The separation properties of each parameter on *Z* are evaluated through an index measuring the intersection of the corresponding conditional densities.

The components of the parameter vector yielding the smaller intersection are candidates to participate in the conditioning set *K*.

### The proposed solution: key steps

#### Computational algorithm

**Fig. 4:**
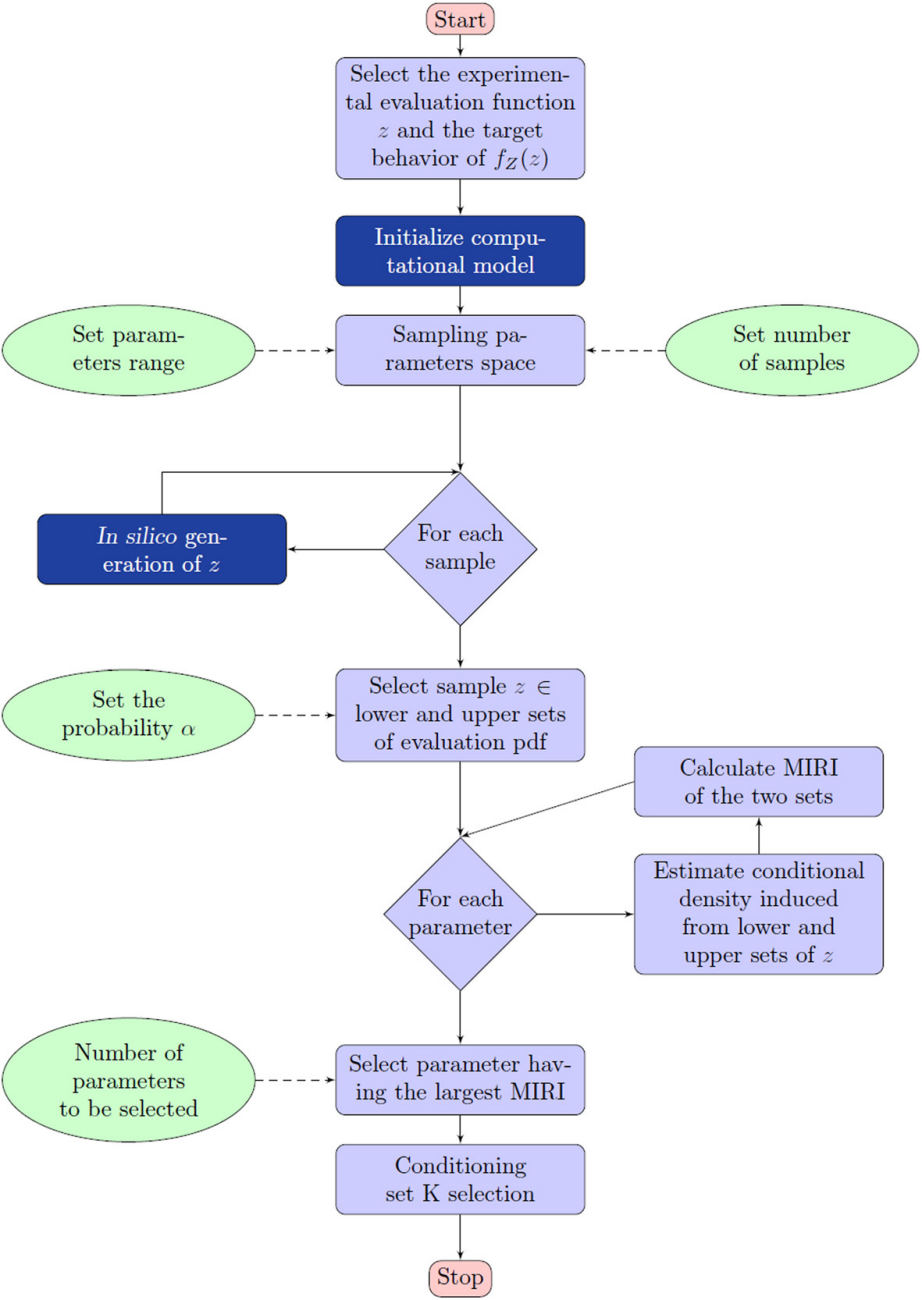
The flowchart of the Conditional Robustness Algorithm (CRA). The CRA has four input values to be set (green ellipses in the diagram) and go through six steps

The entire procedure is visualized in the flowchart in Fig. 4. The Conditional Robustness Algorithm (CRA) has four input values to be set (green ellipses in the diagram): the parameters ranges, the number of samples for the parameters space sampling, the area under sets (4) and the number of selected parameters to design *K*.

The proposed solutions go through the following basic steps (see Fig. 4):

1. **Sampling the parameter space** Generate a sufficiently large set of samples *P*_*S*_ of the parameter vector; i.e., a sufficiently large number of points in the parameter space $\mathbb {P}$.
2. **In silico analysis** For each point *p*∈*P*_*S*_, generate a sample $ z=\zeta ^{\mathcal {S}}_{\tau }{(}{p}{)}$ of the output function by means of numerical simulation of model $\mathcal {S} $, thus building up the set of samples $\mathcal {Z} := \left \{z : z = \zeta ^{\mathcal {S}}_{\tau }{(}{p}{)}, \, \forall p \in P_{S}\right \}$.
3. **Partitioning output sample set**$\boldsymbol{\mathcal {Z}}$ Compute the upper and lower sets *U*=*U*(*α*) and *L*=*L*(*α*), respectively, of set $\mathcal {Z}$, for a given bound *α*.
4. **Estimating conditional densities** For each component *p*_*i*_ of parameter ∈*p*, estimate the two conditional densities $f_{P_{i}|U}(p_{i})$ and $f_{P_{i}|L}(p_{i})$.
5. **Parameter selection** For each parameter component *p*_*i*_∈*p*, compute the moment independent robustness index (defined in the following equation in (7) between the densities $f_{P_{i}|U}(p_{i})$ and $f_{P_{i}|L}(p_{i})$, and select the three parameters having the lowest index values.
6. **Conditioning set selection** Set *K* equal to the three parameters selected above and choose, for each of them, the value corresponding to the maximum of the associated conditional density $f_{P_{i}|L}(p_{i})$ (if one seeks to reduce the evaluation function).

### The proposed solution: step by step discussion

The basic steps outlined above involve a number of ingredients and implications, described in the following.

#### Sampling the parameter space

In this paper, we assume Latin Hypercube Sampling with Linearly spaced samples (L ^2^HS). The use of Latin Hypercube Sampling is common in global approaches to the analysis of complex biochemical networks such as the ones based on Global Sensitivity Analysis. For a comparison of different sampling approaches, see [21] and the references therein.

The issue of linear vs logarithmic sampling is not covered here for the sake of brevity (see [21, 34] for the reasons behind logarithmic sampling).

As for the number of samples, here it has been chosen by looking at the variation of the density *f*_*Z*_(*z*) while increasing the sample number, and stopping once a steady density has been reached.

#### In silico analysis

The computation of the evaluation function, of the model output *z*, is carried out by means of numerical integration of the ODE model $\mathcal {S}$, for each point of the sample set *P*_*S*_, using tools such as Matlab, Octave or similar ones.

#### Partitioning the output sample set $\mathcal {Z}$ and the parameter space *P*_*S*_

According to definitions (4), the set of samples $\mathcal {Z}$ is partitioned and the two subsets *L* and *U* are computed for a given value of design parameter *α*. Based on these subsets, the corresponding subsets of *P*_*S*_ are aggregated:

(6a) $$\begin{array}{@{}rcl@{}} P_{U} & := & \left\{ p \in P_{S} : z = \zeta^{\mathcal{S}}_{\tau}{(}{p}{)} \in U \right\}, \end{array} $$

(6b) $$\begin{array}{@{}rcl@{}} P_{L} & := & \left\{ p \in P_{S} : z = \zeta^{\mathcal{S}}_{\tau}{(}{p}{)} \in L \right\}. \end{array} $$

#### Estimating conditional densities

To estimate the conditional densities $f_{P_{i}|U}(p_{i})$ and $f_{P_{i}|L}(p_{i})$, we use a kernel density approach. Each density is estimated as a superposition of a suitable number of gaussian densities. The non-parametric nature of the approach is of special interest here, as well as its multivariate nature [38–40].

For each component *p*_*i*_ of the parameter vector, we pick up conditioned values from the sets *P*_*U*_ and *P*_*L*_ (i.e., we pick up the values giving rise to output values in the two sets *U* and *L*) and use them to estimate the corresponding conditional densities.

Criteria to evaluate the accuracy of the estimated density turns out to be important whenever the raw data do not show a sufficiently large set of values.

#### Conditioning set selection

For each parameter *p*_*i*_ a weight computed as the intersection between the two densities $f_{P_{i}|U}$ and $f_{P_{i}|L}$ is assigned. More precisely, for each parameter we compute a Moment Independent Robustnesss Indicator (MIRI) *μ*_*i*_ defined as:

(7) $$ \mu_{i} := \int | f_{P_{i}|U} - f_{P_{i}|L}| d p_{i}, \quad i=1, \cdots, q.  $$

The MIRI indicator resembles the moment independent sensitivity indicator *δ* proposed by [41]. While the *δ* indicator is aimed at studying the shift between an unconditional case and a “sensitivity” one, here we compare two “conditioned” cases. Also, here the conditioning events, the lower and upper sets, have fixed probabilities *α*, while the *δ* indicator comprises an expectation over the conditioning variables.

The MIRI is then used to sort the parameter components: the selected ones are those with larger shifts between the two conditional densities, those with higher MIRI values. The conditioning values associated with those selected parameters are the values corresponding to the mode (the maximum of the associate conditional density).

## Results

To verify the performance of the proposed conditional robustness approach, we applied it in two biologically relevant cases. The first example is taken from the field of Synthetic Biology: the incoherent feedforward network called “pulse generator network” [17]. We used this simple example to prove the general application of our approach and also to work with a tractable dimension of the parameter space.

The second example is an application from Cancer Systems Biology [3]: we investigate the robustness of the EGFR IGF1R pathway in lung cancer [20]. This model is directly related to the inspiration of the methodology we proposed in this paper.

### Pulse generator network

**Fig. 5:**
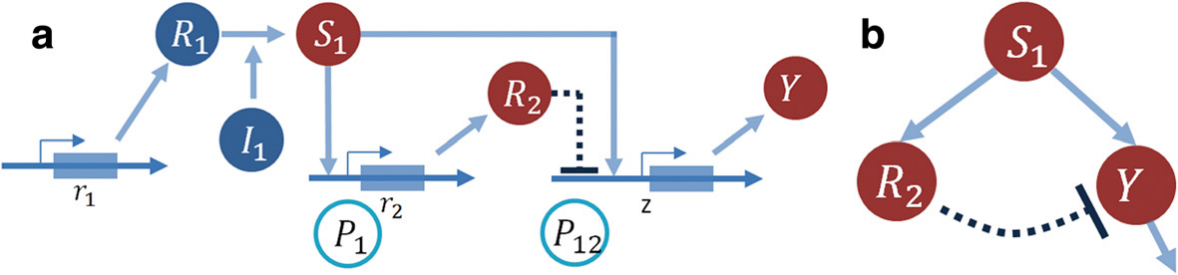
Pulse generator network. **a** Architecture of the pulse-generating network. **b** Equivalent three nodes feedforward network

The pulse generator network is an artificial network used in Synthetic Biology to achieve coordinated behavior in cell communities. The model we use here is derived according to the simplified structure proposed in [42], based on an incoherent feedforward loop network. The system, shown in Fig. 5a, includes an inducing input signal (*S*_1_) activating both the transcription of a reporter gene from a multi-input promoter (*P*_12_) as well as the expression, through the promoter *P*_1_, of a repressor (*R*_2_) of the *P*_12_ promoter ([43], pag. 279). The corresponding simplified model is a three node incoherent feedforward network where the input signal *S*_1_ activates *R*_2_ and the output *Y* and the repressor *R*_2_ deactivates the promoter *P*_12_ and its product *Y* ([42, 43], pag. 279) (see Fig. 5b).

A mathematical model for the topology in Fig. 5b can be obtained using a Hill function for the activation and the repression function [42]. The corresponding ODEs are given by:

(8a) $$\begin{array}{@{}rcl@{}} \dot{R}_{2}& = & k_{1} \frac{(S_{1}/K_{1})^{n_{1}}}{1+(S_{1}/K_{1})^{n_{1}}} -\lambda_{2} R_{2} \end{array} $$

(8b) $$\begin{array}{@{}rcl@{}} \dot{Y} &= & \frac{k_{12}}{1+(R_{2}/K_{2})^{n_{2}}} \frac{(S_{1}/K_{1})^{n_{1}}}{1+(S_{1}/K_{1})^{n_{1}}} - \lambda Y \end{array} $$

where *k*_1_=5 nM/min, *k*_12_=20 nM/min, *λ*_2_=0.01 nM/min, *λ*=0.04 nM/min, *K*_1_=1 nM, *K*_2_=100 nM, and *n*_1_=*n*_2_=3 (see [43]).

**Fig. 6:**
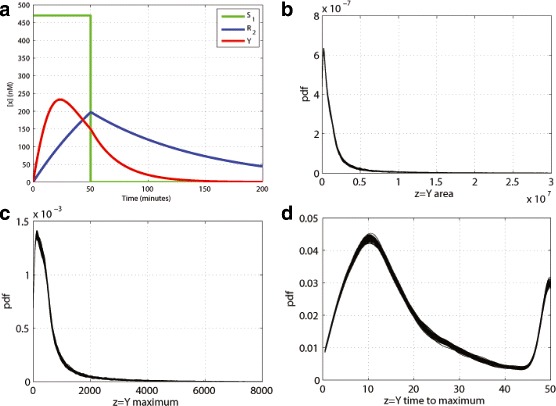
Pulse generator network simulation. **a** Time response of pulse generator network to an input signal with intensity *S*_1_=470 nM and duration 50 min (*k*_1_=5 nM/min, *k*_12_=20 nM/min, *λ*_2_=0.01 nM/min, *λ*=0.04 nM/min, *K*_1_=1 nM, *K*_2_=100 nM, and *n*_1_=*n*_2_=3). **b** 100 realizations of the pdf of the *Y* area; **c** 100 realizations of the pdf of the *Y* maximum. **d** 100 realizations of the pdf of the time to maximum of *Y*

Figure 6a shows the time behavior of the network output *Y* when we apply an inducing rectangular input signal with amplitude equal to *S*_1_=470 nM and duration of 50 min [42]. The model simulation, assuming the wild type values (i.e., nominal) for the parameters, gives an output pulse signal *Y* with the property that the intensity of the pulse, as well as the maximum value and the duration, are not maximized. In Synthetic Biology, the interest is on the possibility to engineer variants of the pulse-generator circuit output exhibiting different quantitative responses such as **increased duration** and **increased intensity** of the pulse.

We applied our approach taking three different evaluation functions for the output function *Y*: the area under the curve, the maximum value and the time of occurrence of the maximum.

We set up *in silico* simulations for the model (8) to generate the measure of the area under the curve of signal *Y* as described in the “Methods” section: we selected the parameter space $\mathbb {P}$ by using as the lower bounds $\frac {1}{10}$ and as the upper ones 10 times the nominal value in $\mathbb {R}_{>0}^{6}$. We fixed the number of sample to *N*=10000 and we generated 100 realizations of the subset *P*_*S*,*i*_,*i*=1,…,100, of the parameters space with *L*^2^*HS*. The kernel distribution estimates for the 100 realizations of the three evaluation functions are shown in Fig. 6b, 6c and 6d, respectively.

For the area of the *Y* signal we applied the proposed algorithm with the goal of selecting the parameters that are most significant to maximize such an indicator. According to the procedure we fixed the *α* parameter for the sets *L* and *U*, as in (4), to *α*=0.1. Then, the kernel distributions were estimated. Additional file 1: Figure S1 shows the upper set distributions $f_{P_{i}|U}(p_{i})$ (red line) and the lower distributions $f_{P_{i}|L}(p_{i})$ (blue line) for the whole parameter set, i.e., *k*_1_, *K*_1_, *k*_12_, *K*_2_, *λ*_2_ and *λ*, for a single realization of *P*_*S*_.

**Fig. 7:**
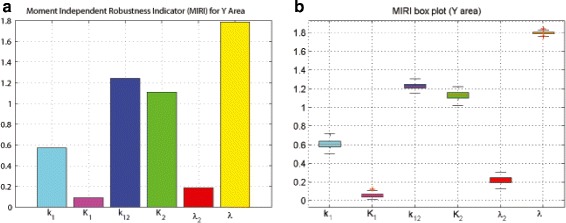
Moment Independent Robustness Indicator (MIRI) for the Y signal area. **a** Single realization. **b** Box plot for 100 realizations

MIRI was evaluated for each parameter (Fig. 7a) and the most significant parameters turn out to be *λ*, *k*_12_ and *K*_2_, as can be inferred, for a single realization of *P*_*S*_, from Additional file 1: Figure S1F, Additional file 1: Figure S1C and Additional file 1: Figure S1D, respectively. MIRI for the six model parameters was evaluated for the 100 realizations and the result is presented in the box plot in Fig. 7b, confirming *λ*, *k*_12_ and *K*_2_ as the parameters most relevant for the pulse area control.

Since we are interested in maximizing the pulse area, we used the parameter densities conditioned on the upper sets, and chose as conditioning values the corresponding modes. Additional file 2: Figure S2 shows the *λ*, *k*_12_ and *K*_2_ lower and upper sets probability density for all the realizations and it confirms the low variability of the probability density shapes over the parameter space sampling.

**Fig. 8:**
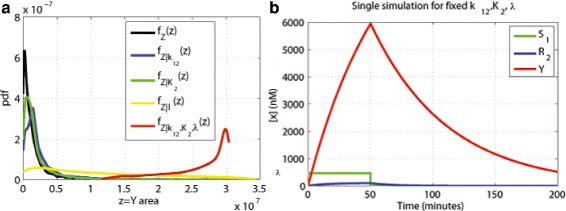
Conditional robustness for Y area (*N*=10000). **a** Probability density function. **b** Simulation examples conditioning parameters as shown in first row of Table 1

Conditional robustness was performed setting each parameter to the values chosen according to the procedure above. Figure 8a shows the conditional probability density estimates setting *k*_12_, *K*_2_ and *λ* parameters as shown in Table 1. Also $f_{Z|k_{12},K_{2},\lambda }(z)$ is compared with the unconditional probability density for the evaluation function. The parameter *λ* plays the major role in maximizing the pulse area and the use of the three parameters selected allows us to obtain the expected behavior. Figure 8b presents an example of simulation where the parameters *k*_12_, *K*_2_ and *λ* are fixed and the other parameters are randomly selected: the network generates a version of the *Y* signal with increased area.

**Table 1:** Parameters setting for the pulse generator and EGFR-IGF1R *in silico* experiments

Experiment	Parameters setting
*Y* area	*k*_12_=174.1101, *K*_2_=789.4512, *λ*=0.0163
*Y* maximum	*k*_12_=177.8501, *λ*=0.0204
*Y* time to maximum	*k*_1_=43.4827, *K*_2_=40.0512,*λ*=0.3360
Multiobjective of *Y*	*k*_12_=177.8501, *K*_2_=40.0512, *λ*=0.0163
*E* *R* *K* ^∗^ activity	*p*_23_=73.9765, *p*_24_=3.9577×10^6^, *p*_35_=23.5795, *p*_36_=6.0595×10^5^, *p*_27_=1.0195

To further test our approach, we performed the robustness analysis using as evaluation function the maximum value reached by the signal *Y*. As shown in Additional file 3: Figure S3, MIRI values for a single realization of the parameters *L*^2^*HS* sampling (Additional file 3: Figure S3A) and MIRI box plot for 100 realization (Additional file 3: Figure S3B) suggested that two parameters are relevant for this evaluation function: *k*_12_ and *λ*.

We fixed *k*_12_ as in Table 1; i.e., the value giving rise to the maximum of the densities conditioned over the upper set and then we performed conditional robustness analysis. The simulation results are shown in Additional file 4: Figure S4A: the blue curve is the conditional density $f_{Z|k_{12}}(z)$: its first moment is higher than the unconditioned distribution *f*_*Z*_(*z*); the yellow curve is the conditional density function, *f*_*Z*|*λ*_(*z*) (see Table 1) and the distribution spread at higher values of the evaluation function; the red line is the *f*_*Z*|*k*12,*λ*_(*z*) and the most probable output value is the one corresponding to the density maximum. An example of the conditioned behavior is showed in Additional file 4: Figure S4B.

For the third evaluation function, the time to maximum of the signal *Y*, *in silico* simulations were performed and MIRI for single realization of the parameter space and 100 realizations are shown in Additional file 5: Figure S5. The key parameters are *k*_1_, *K*_2_ and *λ*.

The parameter values to be used for the conditioning operation are in Table 1 from the lower set parameters distributions. In this case, we are interested in minimizing the time to maximum of the signal *Y*; the results for the analysis are presented in Additional file 6: Figure S6A. The simulation example with the selected values of *k*_1_, *K*_2_ and *λ* confirmed the ability of the proposed procedure to give a ready response of the signal *Y*, that upon conditioning reaches the maximum in 1.5 min (Additional file 6: Figure S6B).

**Fig. 9:**
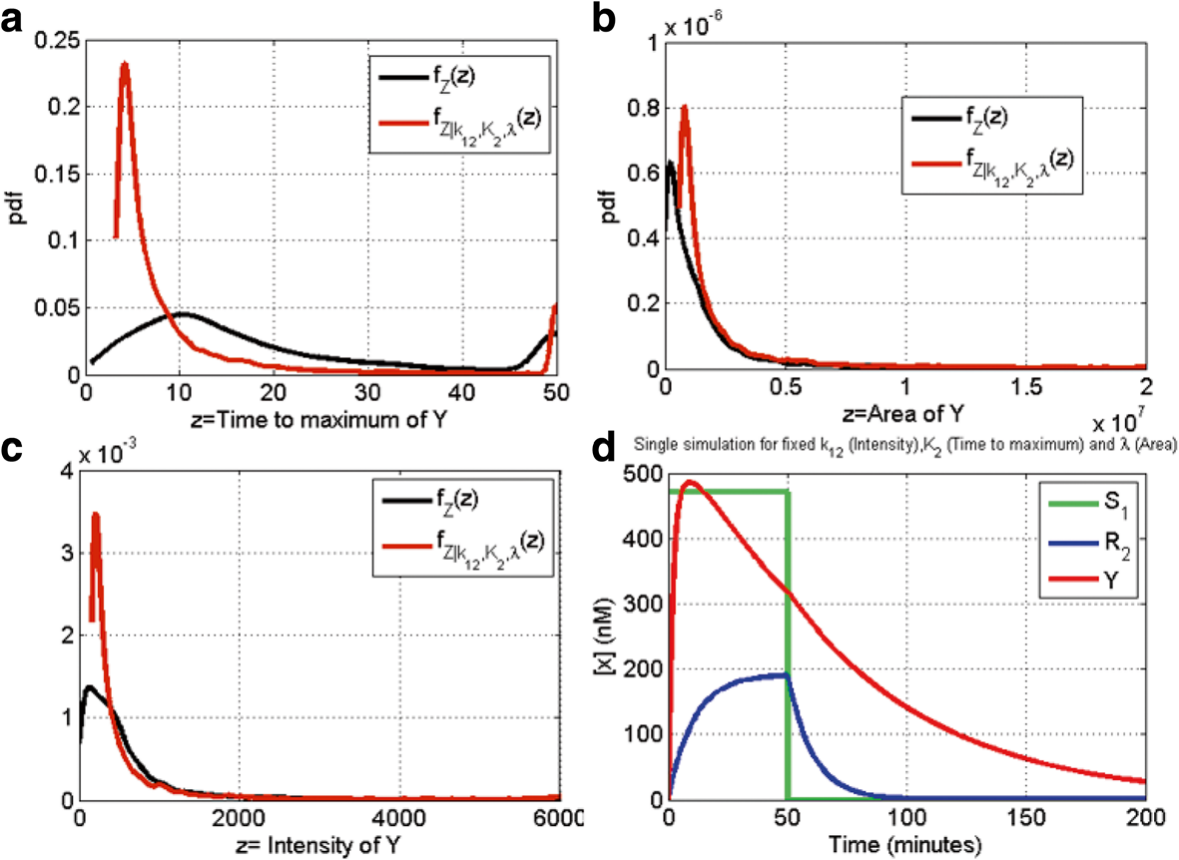
Multiobjective conditional robustness for area, time to maximum and amplification of Y (*N*=10000 and parameters values are shown in the forth row of Table 1). **a** Pdf for time to maximum of Y. **b** Pdf for area of Y. **c** Pdf for Intensity of Y. **d** Simulations example with fixed *k*_12_, *K*_2_ and *λ*

Finally, we performed conditional robustness analysis with the goal of obtaining a fast amplified pulse generation; therefore, we minimized the time to maximum of *Y* and maximized both the area and the intensity of *Y*. For each of the above indexes we chose the parameter with the higher MIRI according to the previous analysis (see Fig. 7b, Additional file 3: Figure S3B and Additional file 5: Figure S5B). *K*_2_ minimizes the time to maximum of *Y* and *k*_12_ and *λ* maximizes the area and the intensity of the pulse *Y*, respectively. The modes are presented in the forth row of Table 1. Figure 9 shows the results of *in silico* experiment through conditional probability densities for the time to maximum of Y (Fig. 9a), the intensity of *Y* (Fig. 9b) and the area of *Y* (Fig. 9c). An example of time behavior of the model states with the selected values for the *k*_12_, *K*_2_ and *λ* parameters is shown (see Fig. 9d).

### EGF-IGF network in lung cancer

**Fig. 10:**
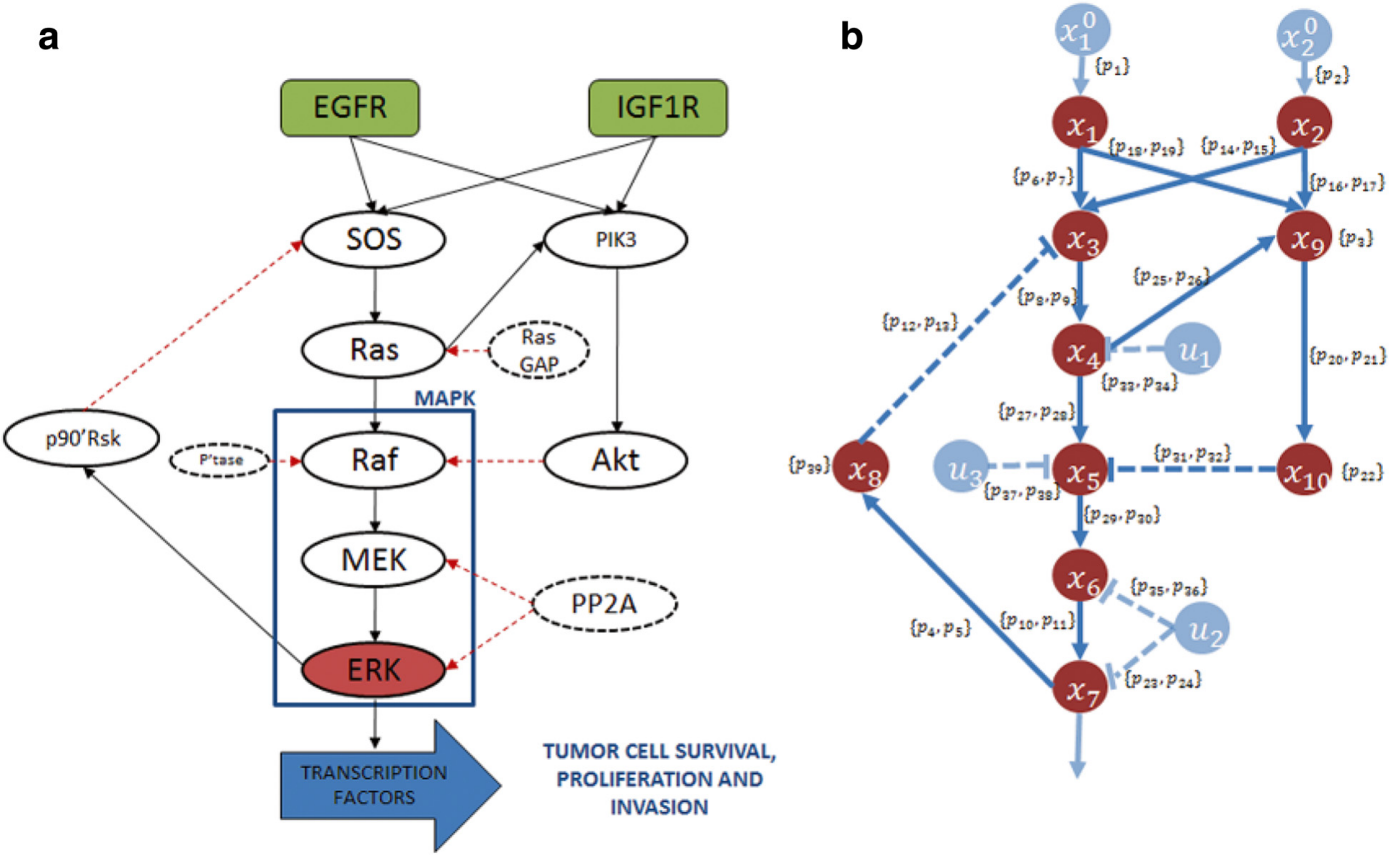
The EGFR-IGF1R pathways in NSCLC. **a** Pathways graph as presented in [46]. **b** State variables representation of the EGFR-IGF1R pathways

The proposed algorithm has been applied to the EGFR-IGF1R network, which is our key motivating example [44]. This network is important in cancer pathogenesis and progression, mostly in the development of Non Small Cell Lung Cancer (NSCLC). Although this signaling pathway is a therapeutic target, recent clinical trials have exhibited limited effects [45]. These complex networks include interactions between epidermal growth factor receptor (EGFR), insulin-like growth factor 1 receptor (IGF1R) along with their downstream effectors of the Mitogen-activated protein kinases (MAPK) cascade and the phospholipids inositol kinases axis (PIK3). One of the main downstream effectors of this network is ERK, a kinase able to phosphorylate both cytosolic and nuclear substrates, including several transcription factors (see pathway in Fig. 10a and [20]).

The biochemical network is sketched in Fig. 10b. The activation of the *EGFR* and *IGF*1*R* (*x*_1_ and *x*_2_, respectively) induced by the ligands binding triggers a cascade of complex proteins interactions that leads to the activation of the *ERK* protein (*x*_7_). There is biological evidence that the time behavior of *ERK* concentration in its active form is strictly related to the proliferation attitude of the cell [11]. The complete model can be found in [20] and [46]. For this system the identification problem has been studied in [47].

Here, we rewrite the model in [20] considering the conservation law and assuming (as is common in this field) that the total amount of each protein and receptor is constant and equal to ${x_{i}^{T}}$, *i*=1,⋯,10, as presented in [48]. The complete system has 10 state variables, 3 input signals and a parameter vector comprising 39 entries. The output function *z* of interest, the evaluation function, is the area under the *ERK*^∗^ curve over the whole time span studied (30 min). This evaluation function is selected because *ERK*^∗^ can be measured with several experimental methodologies such a quantitative immunoblotting, immunofluorescence, in live-cell by means of fluorescence microscopy and mass spectrometry [49] and in patient samples with immunohistochemistry [50]. In [11] the measure of ERK concentration is used to infer the silencing of its activity in tumor cells.

$$ \begin{array}{rcl} \dot{x}_{1} \!& \!= \! &\! -p_{1}\,{x_{1}}\\ \dot{x}_{2} \!& \!= \! &\! -p_{2}\,{x_{2}}\\ \dot{x}_{3} \!& \!= \! &\! p_{6}{x_{1}}\frac{{{x^{T}_{3}}} - {x_{3}}}{p_{7}+{{x^{T}_{3}}} - {x_{3}}} + p_{14}{x_{2}}\frac{{{x^{T}_{3}}} - {x_{3}}}{p_{15}+{{x^{T}_{3}}} - {x_{3}}} \\ &&- p_{12}{x_{13}}\frac{{x_{3}}}{p_{13}+{x_{3}}} \\ \dot{x}_{4} \!& \!= \! &\! p_{8}{x_{3}}\frac{{{x^{T}_{4}}}-{x_{4}}}{p_{9}+{{x^{T}_{4}}}-{x_{4}}} - p_{33}{u_{3}}\frac{{x_{4}}}{p_{34}+{x _{4}}}\\ \dot{x}_{5} \!& \!= \! &\! p_{27}{x_{4}}\frac{{{x^{T}_{5}}}-{x_{5}}}{p_{28}+{{x^{T}_{5}}}-{x_{5}}} - p_{37}{u_{1}}\frac{{x_{7}}}{p_{38}+{x_{7}}} \\ && -p_{31}{x_{10}}\frac{{x_{5}}}{p_{32}+{x_{5}}}\\ \dot{x}_{6} \!& \!= \! &\! p_{29}{x_{5}}\frac{{{x^{T}_{6}}}-{x_{6}}}{p_{30}+{{x^{T}_{6}}}-{x_{6}}} - p_{35}{u_{2}}\frac{{x_{6}}}{p_{36}+{x_{6}}}\\ \dot{x}_{7} \!& \!= \! &\! p_{10}{x_{6}}\frac{{{x^{T}_{7}}}-{x_{7}}}{p_{11}+{{x^{T}_{7}}}-{x_{7}}} - p_{23}{u_{2}}\frac{{x_{7}}}{p_{24}+{x_{7}}}\\ \dot{x}_{8} \!& \!= \! &\! p_{4}{x_{7}}\frac{{{x^{T}_{8}}}-{x_{8}}}{p_{5}+{{x^{T}_{8}}}-{x_{8}}} - p_{39}{x_{8}}\\ \dot{x}_{9} \!& \!= \! &\! p_{25}{x_{4}}\frac{{{x^{T}_{9}}}-{x_{9}}}{p_{26}+{{x^{T}_{9}}}-{x_{9}}}+ p_{16}{x_{2}}\frac{{{x^{T}_{9}}}-{x_{9}}}{p_{17}+{{x^{T}_{9}}}-{x_{9}}}\\ &&+ p_{18}{x_{1}}\frac{{{x^{T}_{9}}}-{x_{9}}}{p_{19}+{{x^{T}_{9}}}-{x_{9}}}-p_{3}{x_{9}}\\ \dot{x}_{10} \!& \!= \! &\! +p_{20}{x_{9}}\frac{{x^{T}_{10}}-{x_{10}}}{p_{21}+{x^{T}_{10}}-{x_{10}}}-p_{22}{x_{10}}\\ \end{array}  $$

Following an approach widely used in literature, we assume that each element *p*_*i*_ of the parameter vector *p*, $p \in \mathbb {R}^{39}$, can assume values in a range defined by two multiplicative bounds w.r.t. a nominal (wild-type) value *p*_*wt*,*i*_: a lower bound *b*_*ℓ*,*i*_=*c*_*ℓ*,*i*_*p*_*wt*,*i*_ and an upper bound *b*_*u*,*i*_=*c*_*u*,*i*_*p*_*wt*,*i*_, where the coefficients *c*_*ℓ*,*i*_ and *c*_*u*,*i*_ are the multiplicative perturbations, *c*_*ℓ*,*i*_<1, ∀*i* and *c*_*u*,*i*_<1, ∀*i*. Hence, the parameter space $\mathbb {P}$ is given by the cartesian product $\mathbb {P} =\prod _{i=1}^{39}[c_{\ell,i} p_{wt,i}, \, c_{u,i} p_{wt,i}]$.

The key parameters characterizing the ODE model of a biochemical network are activation rates and Michaelis-Menten constants. In Table Additional file 7: Table S1, the Michaelis-Menten constants are all those whose names start with *KM*. While sampling the parameter space, we will consider the whole parameter vector with 39 entries, since the relationship between activation rates and Michaelis-Menten constants allows us to check the consistency of the analysis. We also assume that the total mean values of the number of molecules in the cell does not change significantly in NSCLC when compared to normal cells (Additional file 8: Table S2 Table).

**Fig. 11:**
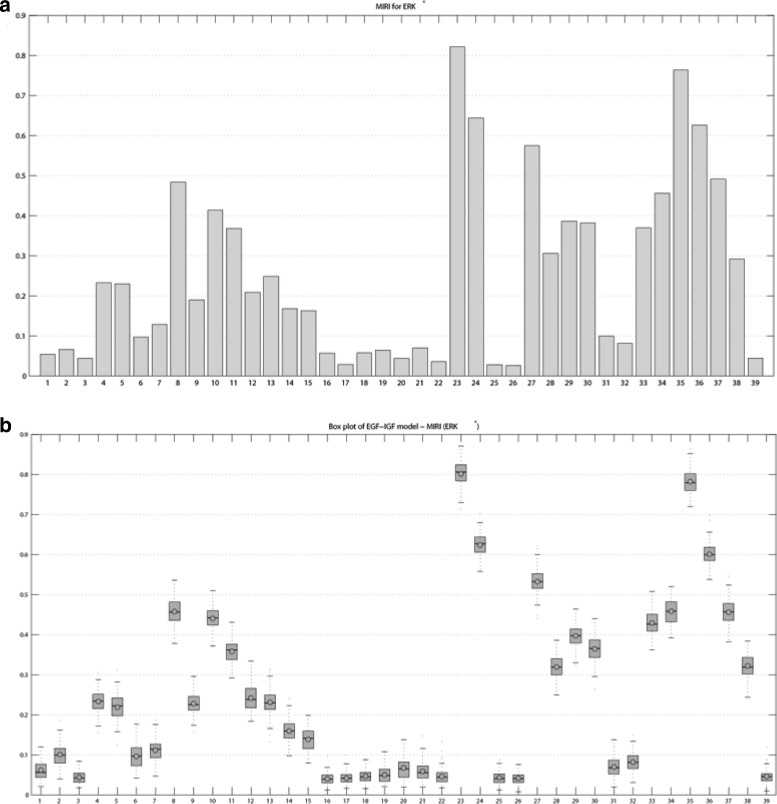
Moment Independent Robustness Indicator (MIRI) for ERK activity in EGFR-IGF1R model. **a** Single realization. **b** Box plot for 100 realizations

We apply conditional robustness analysis for the model in (9) generating *in silico* measures of the active *ERK*. The parameter space $\mathbb {P}$ was sampled with the *L*^2^*HS* method, the number of samples and the number of realizations of the parameter space sampling were fixed to *N*=10000 and 100, respectively. Figure 11a shows a realization of MIRI for the EGF-IGF model. The highest MIRI values are achieved by *p*_23_, *p*_24_, *p*_35_ and *p*_36_: they represent the activation rates and the Michealis-Menten constants of protein phosphatase 2A (PP2A) with *ERK* and MAPK/ERK kinase (MEK), respectively. The activation rate of Ras and Raf interaction (*p*_27_) also has relevant MIRI. The MIRI box plot for 100 realizations confirmed the MIRI ordering for the 39 parameters (Fig. 11b).

**Fig. 12:**
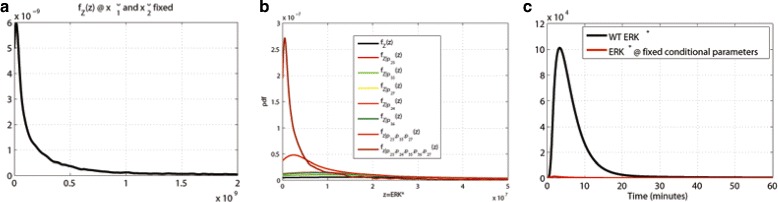
**a** Unconditional distribution of evaluation function *E* *R* *K* ^∗^. **b** Conditional robustness for ERK activity (*N*=10000). **c** *E* *R* *K* ^∗^ time simulation: wild type (black line) vs simulation at conditioned value as in Table 1

The estimated kernel density for the values of the area under *ERK*^∗^ signal is shown in Fig. 12a: it has a mean, a variance and a mode presented in Table 2. We evaluate the conditional robustness for the single value *p*_23_, *p*_24_,*p*_35_, *p*_36_ and *p*_27_ obtained fixing the lower *L* and upper *U* set with a parameter *α*=0.1, and setting them to the highest density values for the corresponding densities conditioned on set *L*, (see last line in Table 1). We evaluate conditional robustness fixing only the activation rates *p*_23_, *p*_35_ and *p*_27_ and also fixing all 5 parameters selected. Figure 12b shows the conditional densities for $f_{Z|p_{23}}(z)$, $f_{Z|p_{35}}(z)$, $f_{Z|p_{27}}(z)$, $f_{Z|p_{24}}(z)$, $f_{Z|p_{36}}(z)$, $f_{Z|p_{23},p_{35},p_{27}}(z)$ and $f_{Z|p_{23},p_{24},p_{35},p_{36}p_{27}}(z)$ and Table 2 presents the statistical descriptors for the conditional densities. The lowest mean, variance are obtained conditioning all the selected parameters. Figure 12c shows a comparison between the wild type simulation of ERK activity (black line) and the conditioned simulation with parameters with fixed value as in Table 1 (red line). The *ERK*^∗^ activity is strongly reduced as showed in Table 2.

**Table 2:** Proliferation output pdf measures

Pdf	Mean	Variance	Mode
*f*_*Z*_(*z*)	2.6×10^8^	1.5805×10^17^	2.1×10^7^
$f_{Z|p_{23}}(z)$	1.5824×10^8^	7.6840×10^16^	1.0×10^7^
$f_{Z|p_{35}}(z)$	1.6075×10^8^	7.0478×10^16^	9.6×10^6^
$f_{Z|p_{27}}(z)$	1.6380×10^8^	9.5956×10^16^	7.2×10^6^
$f_{Z|p_{24}}(z)$	1.3436×10^8^	6.6212×10^16^	7.085×10^6^
$f_{Z|p_{36}}(z)$	1.4505×10^8^	7.0479×10^16^	7.41×10^6^
$f_{Z|p_{23},p_{35},p_{27}}(z)$	4.2358×10^7^	8.0128×10^15^	2.528×10^6^
$f_{Z|p_{23},p_{24},p_{35},p_{36},p_{27}}(z)$	8.2938×10^6^	5.3817×10^14^	5.131×10^5^
*f*_*Z*_(*z*) @ ${x_{1}^{0}}$ and ${x_{2}^{0}}$ perturbed	3.2996×10^8^	2.0223×10^17^	2.451×10^7^
$f_{Z|p_{23},p_{24},p_{35},p_{36}p_{27}}(z)$ @ ${x_{1}^{0}}$ and ${x_{2}^{0}}$ perturbed	1.3099×10^7^	1.4868*e*×10^15^	2.528×10^6^

**Fig. 13:**
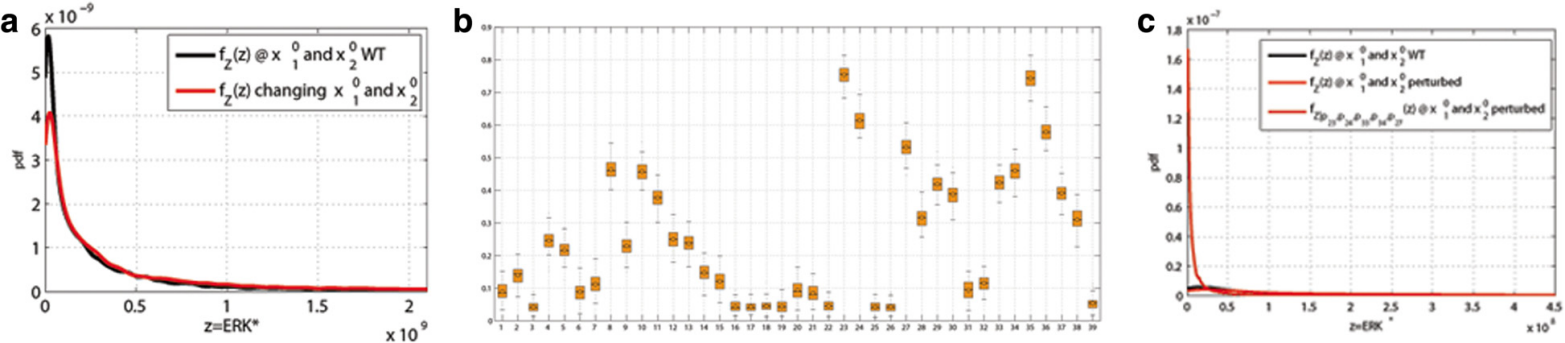
**a** Unconditional *E* *R* *K* ^∗^ activity at *EGFR* and *I* *G* *F*1*R* initial condition wild type and at perturbed *EGFR* and *I* *G* *F*1*R*. **b** MIRI of the parameters for 100 realizations. **c** Effect of conditioning of *p*_23_, *p*_24_,*p*_35_, *p*_36_ and *p*_27_

It is known from biochemical and clinical literature that the concentration of the receptors *EGFR* and *IGF*1*R* has a major role in the proliferation attitude (see, among others, [20, 44] and the references therein). To exploit this characteristic, we also investigated perturbation on the initial value of ${x_{1}^{0}}=EGFR$ and ${x_{2}^{0}}=IGF1R$. We use a receptor space *X*_1,2_, obtained in a manner similar to $\mathbb {P}$, i.e., ${X}_{1,2} := \left [c_{\ell,1}x^{0}_{wt,1}, \,c_{u,1}x_{wt,1}1^{0}\right ]\,\times \, \left [c_{\ell,2}x_{wt,2}^{0}, \,c_{u,2}x_{wt,2}^{0}\right ]$, where the *c*’s coefficients are the perturbations and $x_{wt,i}^{0}$, *i*=1,2, are the wild-type initial conditions. Figure 13a shows the unconditioned measure distribution with perturbed *EGFR* and *IGF*1*R* (red line) that shows higher mean and variance than is the case with fixed initial conditions (see Table 2). The MIRI box plot in Fig. 13b, generated with our algorithm by perturbing both the 39 parameters and the ${x_{1}^{0}}$ and ${x_{2}^{0}}$ initial conditions, confirmed the same conditioning set obtained in the previous analysis. Conditioning the *p*_23_, *p*_24_,*p*_35_, *p*_36_ and *p*_27_ parameters we are still able to reduce the mean and variance (see Table 2 and Fig. 13c)).

## Discussion and Conclusions

We have presented a novel approach to study the robustness of complex biological networks and discover their fragility. We have used this approach as a base to design a new algorithm aimed at selecting a few of the system parameters that would allow us to achieve desired conditional robustness properties. The approach is based on the combination of robustness analysis, moment independent robustness indicator, and estimated kernel conditional densities. The approach takes its motivation from Cancer Systems Biology and the associated problem of drug therapy strategies, namely the selection of crucial proteins that need to be inhibited with the administration of therapeutic compounds with the goal to block cancer proliferation and progression [3]. Nevertheless, the proposed algorithm is general in nature and its suitability for a larger class of problems has been shown by means of a case study from Synthetic Biology.

Our algorithm is similar to Global Sensitivity Analysis (GSA) approaches as far as the sampling of the parameter space is concerned, while it is different from the point of view of the goals of analysis. Both GSA and our Conditional Robustness Algorithm (CRA) have to deal with the sampling of a large parameter space. Key approaches to this issue are Sobol Low Discrepancy Sequence, Monte Carlo techniques, Latin Hypercube and variants of all of the above. Here, we use Latin Hypercube sampling; additional results (data not shown) show that Sobol techniques yield similar results. Sampling techniques are discussed in [21, 23, 34] and the references therein. Uniform/linear vs logarithmic distribution is another relevant issue when sampling of large spaces has to be performed. The “priori” knowledge on parameter variability is related to to this issue. See [21, 34] for a more detailed discussion. In this paper, we focus on Latin Hypercube Sampling with Linearly spaced samples (*L*^2^*HS*) since our focus is on the key properties of the proposed algorithm.

As for the main goal of our approach, we are seeking a strategy to identify regions of the parameter space yielding desired system behavior. On the contrary, most of the GSA results are finalized to investigate the sensitivity of key performance indices with respect to variation on system parameters, usually in the classical form of derivative of the index with respect to parameters. Hence, GSA methods are quite useful as analysis tools and they provide “results that could potentially help in the optimization of the system” [27]. Similarly, they do not yield specific design criteria to enforce given system capabilities, as with this problem we are investigating on the problem we are investigating. Here our main interest is on the selection of some parameters and their values to achieve a desired behavior, and not on the deduction of the parameters having the largest influence on a given performance function.

We have shown how the CRA tool can be used both in Cancer Systems Biology and in Synthetic Biology through two examples, the *EGFR*- *IGF*1*R* network in non small cell lung cancer and a reduced model of pulse generator network.

The development of computational tools to study cancer disease is an emergent field in Cancer Systems Biology. The general formulation of robustness proposed in [36] and its extensions to drug development in cancer research [9] were explored in our paper by introducing the idea of conditional robustness.

Our approach assumes the knowledge of a mathematical model of the biological system and the associated parameter space. In addition, a proper evaluation function whose behavior over the whole parameter space can be described by means of an associated density function must be defined. The problem we are interested in, namely the Conditional Robustness Problem, is that of shifting such a density to a desired region in order to achieve the suitable global behavior.

The mathematical model used here is based on ordinary differential equations; nevertheless, several others types of mathematical tools, e.g. stochastic models, boolean models and many other, can be used as well with minor modification. The dependence of the Conditional Robustness Algorithm on the specific modeling approach is confined to the two blocks in dark blu in Fig. 4. More specifically, the key condition is the availability of a computable relationship between system parameters and evaluation function. Notice that both numerical and analytical maps can be used. From a mathematical point of view, any modeling approach allowing to compute the input-output map in (2) can be directly cast in our general framework. As an example, the Boolean model in [51] has been implemented by the authors through a proper simulation model, which depends on a number of rules, initial states and other parameters. Each execution of the block “in silico generation of *ζ*” in our CRA, for a given parameter configuration, turns out to be a corresponding simulation of the above boolean model.

*In silico* experiments are used to generate the density function describing the property of interest as well as to estimate the effect on such density of the parameter conditioning, measuring the amount of overlapping between the parameters conditioned density functions. From a technical point of view, the distance between the parameter densities is a separation measurement in the sense of [52], and it is evaluated trough the Moment Independent Robustness Index (MIRI) which is inspired to the measure introduced in [41]. Borgonovo et al. illustrated that variance is not always sufficient enough to describe uncertainty and pointed out that a sensitivity measure should refer to the entire output distribution instead of a particular moment. Therefore we introduce in our algorithm the MIRI. A higher value of the MIRI indicates a greater separation between the densities and a greater capacity of the parameter to discriminate between different behaviors of the evaluation function. The parameters with largest MIRI are selected as the conditioning ones, and their values are fixed to the modes of the conditional densities, allowing to achieve the desired system behavior.

The analysis of the EGFR-IGF1R model in non small cell lung cancer is an example of nontrivial biochemical network in cancer application. The output of the EGFR-IGF1R network is the active form of the ERK protein, which is related to the cancer proliferation. We considered the area under the active ERK curve as a measure of proliferation, and our goal is to reduce this network output almost to zero, namely to silence the proliferation activity.

In [11] the authors concluded that cell proliferation is effectively silenced only when active ERK protein level falls below a lower threshold. Their findings provide rationale for combined inhibition of multiple nodes in the ERK pathway, since acting on a single node turns out not to be enough to constrain ERK signal below the threshold required for a proper proliferation level. A multiple node action is the objective of the computational algorithm we are proposing, whose goal is finding to how to silence ERK protein with a conditioning action on multiple model parameters.

The CRA results suggest three points in the network that could be potentials targets for the inhibition of cancer cell proliferation: the activation rate of RAF/Ras in the MAPK cascade, and the activation rate and Michaelis Menten constants of PP2A both with MEK and ERK. *In silico* experiments show that ERK silencing is more effective whenever all the three nodes are conditioned (by fixing the corresponding five parameters, see Table 1), i.e., they are candidate that could be targeted.

The therapeutic strategies recently proposed, primarily pertaining to co-targeting EGFR and IGF1R receptors, exhibited notable advantages in overcoming resistance to standard chemotherapy. Furthermore, these techniques might offer benefits beyond the limited effects of single-agent targeting previously reported. The strategies of blocking these pathways in combination with the result obtained from our conditional robustness, suggest a novel potential approach to develop future and more effective therapies for cancer treatments [53].

It is well known that the over expression of both EGFR and IGR1R plays a crucial role in the proliferation of cancer cells [44]. The increase in the mean value and variance of proliferation index associated with an increase on the number of receptors showed in this study confirm their role in cancer (see Table 2 and Fig. 13b).

The results obtained can be applied to other problems concerned with drug development. For example, if we have a set of drug candidates that target proteins in a pathway, our methods can be used to asses which is the best compound to use as a single agent or in combination. Figure 12b can be interpreted as the best combination of target to be used and Fig. 13b predicts the ability of the target to silence ERK activity.

We considered the pulse generator network from Synthetic Biology to study the validity of CRA in different frameworks. This analysis allows us to conclude that the algorithm is able to select the proper parameters in order to maximize the pulse transferring (and intensity of the pulse) and minimize the time to reach the pulse maximum. The example also demonstrates the solution for a multi objective evaluation function. For this type of synthetic networks, the knowledge of the most significant parameters and the corresponding values allowing achievement of specific input/output behaviors is of crucial relevance to characterize the biological circuits and is a key piece of information contained in their data sheet.

The algorithm proposed in this paper has four classes of configuration data: the multiplicative perturbation constants *c*_*ℓ*,*i*_ and *c*_*u*,*i*_ defining the parameter space; the number *N* of samples to be taken in the parameter space and used to run *in silico* experiments to compute the corresponding set of samples $\mathcal {Z}$ of the evaluation function; the probability *α* defining the subsets of $\mathcal {Z}$ used to carry out conditional robustness; and the number of parameters to be fixed by the conditioning operation. The selection of those inputs is a user choice and depends on the problem at hand. Below we give some guidelines to select proper values.

In the framework of mathematical modeling of biological systems, and in particular Computational Biology, the nominal system parameters can be either estimated from experimental data with a given range of uncertainty, (and quite often the experiments are carried out in different settings and conditions, or derived using biochemical/biological *a priori* knowledge of the system, and therefore only the order of magnitude can be set). In this setting, a strategy widely used in the literature is to assume that the parameters space spans for two orders of magnitude below and above the nominal value [21, 27, 34].

The choice of the number *N* of samples in the parameters space and the probability *α* defining the extremal subsets *U* and *L* are somehow interdependent. The number *N* of samples has to be set in order to guarantee that the estimated output density function *f*_*Z*_(*z*), as a function of increasing *N*, has reached a steady state shape [34]. The subsets *L* and *U* build upon this distribution generate data used to estimate the conditional densities $f_{P_{i}|L}(p)$ and $f_{P_{i}|U}(p)$ of each parameter. To generate a satisfactory estimation of these densities at least 1.000 samples are required. Hence, on the average, a good estimate for a lower bound on the number *N* of overall samples is equal to $\frac {1.000}{\alpha }$. As an example, in the EGFR-IGFR model we fixed *α*=0.1 and we generated *N*=10.000 samples in the parameter space. Notice that, in this case, such a value is quite higher than the number of samples required to achieve a satisfactory (i.e., stationary with respect to *N*) density *f*_*Z*_(*z*); hence we are over-sampling the parameter space.

The fourth configuration constant of the CRA algorithm is the number of parameters to be fixed, i.e., to be conditioned. Such a number can be 6set by trial and error, looking both to the number of densities $f_{P_{i}|L}(p)$ and $f_{P_{i}|U}(p)$ that turn out to be sufficiently different and to the overall effect on the conditional density *f*_*Z*|*K*_(*z*).

The computational cost of our CRA has two main sources: the *L*^2^*HS* sampling of the parameter space and the ODE numerical integration of the whole set of samples. As for the former, in [54] the computational cost of Matlab function *lhsdesign*, is compared with other optimization techniques with respect to the number of parameters and sampled points. As for the latter, the computational cost associated with the numerical integration of a single ODE depends heavily on model dimensions and model properties, such as its stiffness. Parallel implementations, such as CUDA models, can be used to deal with this issue.

We implemented a parallel versions of our algorithm: referring to the diagram in Fig. 4, the most significant computation cost is the *in silico* data generation associated with the block “For each sample”, which integrates the ODE model. Each ODE integration is independent of the others and therefore they can be executed in parallel on a cluster of work-stations: on a 7 core processor the computation time to generate the set of samples $\mathcal {Z}$ can be reduced by a factor of 7.

In conclusion, our Conditional Robustness Algorithm is a new method to study the role of key parameters to discover the system robustness/fragility or in conditioning the systems to the wished behaviour. The CRA significantly contributes to formal methods in computational systems biology and introduces a new framework to identify target identification and to support drug discovery in oncology.

## Additional files

Additional file 1
**Figure S1.** Kernel density estimation of upper and lower density for the parameters. (A) *k*_1_. (B) *K*_1_. (C) *k*_12_. (D) *K*_2_. (E) *λ*_2_. (F) *λ*. (EPS 1966.08 kb)

Additional file 2
**Figure S2.** Kernel distributions for 100 realizations. (A) *λ*. (B) *k*_12_. (C) *K*_2_. (EPS 1228.8 kb)

Additional file 3
**Figure S3.** Moment Independent Robustness Indicator (MIRI) for the maximum Y. (A) Single realization. (B) Box plot for 100 realizations. (EPS 1126.4 kb)

Additional file 4
**Figure S4.** Conditional robustness for Y signal maximum (*N*=10000). (A) Densities. (B) Simulation examples conditioning parameters as shown in second row of Table 1. (EPS 1157.12 kb)

Additional file 5
**Figure S5.** Moment Independent Robustness Indicator (MIRI) for time to maximum of Y. (A) Single realization. (B) Box plot for 100 realizations. (EPS 1105.92 kb)

Additional file 6
**Figure S6.** Conditional robustness for time to maximum of Y (*N*=10000). (A) Densities. (B) Simulation examples conditioning parameters as shown in third line of Table 1. (EPS 1146.88 kb)

Additional file 7
**Table S1.** EGFR-IGF1R model parameters. (PDF 68.9 kb)

Additional file 8
**Table S2.** Initial concentration of model species and total protein amount. (PDF 68.9 kb)
