# Erratum to: ‘Conditional robustness analysis for fragility discovery and target identification in biochemical networks and in cancer systems biology’

**DOI:** 10.1186/s12918-016-0267-2

**Published:** 2016-03-02

**Authors:** Fortunato Bianconi, Elisa Baldelli, Vienna Ludovini, Emanuel F. Petricoin, Lucio Crinò, Paolo Valigi

**Affiliations:** Department of Experimental Medicine, University of Perugia, Polo Unico Sant’Andrea delle Fratte, Via Gambuli, 1, 06156 Perugia, Italy; Center for Applied Proteomics and Molecular Medicine George Mason University, 10900 University Blvd, 20110 Manassas, USA; Department of Medical Oncology, Santa Maria della Misericordia Hospital, Azienda Ospedaliera di Perugia, Piazzale Menghini, 1, Loc. Sant’Andrea delle Fratte, 06156 Perugia, Italy; Department of Engineering, University of Perugia, G. Duranti, 93, 06125 Perugia, Italy

Unfortunately, the original version of this article [1] contained an error. The spelling of the author [Vienna Luovini,] name was incorrect. This has been corrected above and also included correctly below:

Vienna Ludovini
