# Supplementary material for: Conditional robustness analysis for fragility discovery and target identification in biochemical networks and in cancer systems biology
Source: BMC Syst Biol. 2015 Oct 19;9:70. doi: 10.1186/s12918-015-0216-5 (PMC4617482; doi:10.1186/s12918-015-0216-5)
Supplement: Additional file 7 — Table S1. EGFR-IGF1R model parameters. (PDF 68.9 kb) [file 12918_2015_216_MOESM7_ESM.pdf]

Additional file 7: Table S1

EGFR-IGF1R model parameters.

Parameter	Name	Value
p_1	γ_{EGFR}	0.02
p_2	γ_{IGF1R}	0.02
p_3	kd_{PIK3*}	0.005
p_4	$k_{p90Rsk:Erk}$	0.0213697
p_5	$KM_{p90Rsk:Erk}$	763523.0
p_6	$k_{SOS:E}$	694.731
p_7	$KM_{SOS:E}$	6086070.0
p_8	$k_{Ras:SOS}$	32.344
p_9	$KM_{Ras:SOS}$	35954.3
p_{10}	$k_{Erk:MEK}$	9.85367
p_{11}	$KM_{Erk:MEK}$	1007340.0
p_{12}	$k_{DSOS:p90Rsk}$	161197.0
p_{13}	$KM_{DSOS:p90Rsk}$	896896.0
p_{14}	$k_{SOS:I}$	500.0
p_{15}	$KM_{SOS:I}$	1000000.0
p_{16}	$k_{PIK3:IGF1R}$	10.6737
p_{17}	$KM_{PIK3:IGF1R}$	184912.0
p_{18}	$k_{PIK3:EGFR}$	10.6737
p_{19}	$KM_{PIK3:EGFR}$	184912.0
p_{20}	$k_{Akt:PIK3}$	0.0566279
p_{21}	$KM_{Akt:PIK3}$	653951.0
p_{22}	kd_{Akt}	0.0050
p_{23}	$k_{Erk:PP2A}$	9.85367
p_{24}	$KM_{Erk:PP2A}$	1007340.0
p_{25}	$k_{PIK3:Rsa}$	0.0771067
p_{26}	$KM_{PIK3:Rsa}$	272056.0
p_{27}	$k_{Raf:Ras}$	0.884096
p_{28}	$KM_{Raf:Ras}$	62464.6
p_{29}	$k_{Raf:MEK}$	185.759
p_{30}	$KM_{Raf:MEK}$	4768350.0
p_{31}	$k_{Raf:Akt}$	15.1212
p_{32}	$KM_{Raf:Akt}$	119355.0
p_{33}	$k_{Ras:RasGab}$	1509.36
p_{34}	$KM_{Ras:RasGab}$	1432410.0
p_{35}	$k_{MEK:PP2A}$	2.83243
p_{36}	$KM_{MEK:PP2A}$	518753.0
p_{37}	$k_{Raf:RafPP}$	0.126329
p_{38}	$KM_{Raf:RafPP}$	1061.71
p_{39}	kd_{p90Rsk}	0.0050
