# Supplementary material for: Conditional robustness analysis for fragility discovery and target identification in biochemical networks and in cancer systems biology
Source: BMC Syst Biol. 2015 Oct 19;9:70. doi: 10.1186/s12918-015-0216-5 (PMC4617482; doi:10.1186/s12918-015-0216-5)
Supplement: Additional file 8 — Table S2. Initial concentration of model species and total protein amount. (PDF 68.9 kb) [file 12918_2015_216_MOESM8_ESM.pdf]

Additional file 8: Table S2

Initial concentration of model species and total protein amount

Variable	Species	Value
x_1	$EGFR^*$	8000
x_2	$IGF1R^*$	650
x_3	SOS	0
x_4	Ras^*	0
x_5	Raf^*	0
x_6	MEK^*	0
x_7	Erk^*	0
x_8	$p90Rsk^*$	0
x_9	$PIK3^*$	0
x_{10}	Akt^*	0
u_1	$RafPP$	120000
u_2	$PP2A$	120000
u_3	$RasGab$	120000
x_3^T	$DSOS$	120000.0
x_4^T	Ras	120000
x_5^T	Raf	120000
x_6^T	MEK	600000
x_7^T	Erk	600000
x_8^T	$p90Rsk$	120000
x_9^T	$PIK3$	120000
x_{10}^T	Akt	120000
